# Prognostic Impacts of Angiopoietins in NSCLC Tumor Cells and Stroma: VEGF-A Impact Is Strongly Associated with Ang-2

**DOI:** 10.1371/journal.pone.0019773

**Published:** 2011-05-16

**Authors:** Sigve Andersen, Tom Donnem, Khalid Al-Shibli, Samer Al-Saad, Helge Stenvold, Lill-Tove Busund, Roy M. Bremnes

**Affiliations:** 1 Institute of Clinical Medicine, University of Tromso, Tromso, Norway; 2 Department of Oncology, University Hospital of North Norway, Tromso, Norway; 3 Institute of Medical Biology, University of Tromso, Tromso, Norway; 4 Department of Pathology, Nordland Central Hospital, Bodo, Norway; 5 Department of Pathology, University Hospital of North Norway, Tromso, Norway; University Health Network, Canada

## Abstract

**Introduction:**

Angiopoietins and their receptor Tie-2 are, in concert with VEGF-A, key mediators in angiogenesis. This study evaluates the prognostic impact of all known human angiopoietins (Ang-1, Ang-2 and Ang-4) and their receptor Tie-2, as well as their relation to the prognostic expression of VEGF-A.

**Methods:**

335 unselected stage I-IIIA NSCLC-patients were included and tissue samples of respective tumor cells and stroma were collected in tissue microarrays (TMAs). Immunohistochemistry (IHC) was used to semiquantitatively evaluate the expression of markers in duplicate tumor and stroma cores.

**Principal Findings:**

In univariate analyses, low tumor cell expression of Ang-4 (P = 0.046) and low stromal expressions of Ang-4 (P = 0.009) and Ang-2 (P = 0.017) were individually associated with a poor survival. In the multivariate analysis, low stromal Ang-2 (HR 1.88; CI 95% 1.15-3.08) and Ang-4 (HR 1.47, CI 95% 1.02–2.11, P = 0.04) expressions were independently associated with a poor prognosis. In patients with high tumor cell expression of Ang-2, a concomitantly high tumor VEGF-A expression mediated a dramatic survival reduction (P<0.001). In the multivariate analysis of patients with high Ang-2 expression, high tumor VEGF-A expression appeared an independent poor prognosticator (HR 6.43; CI 95% 2.46–16.8; P<0.001).

**Conclusions:**

In tumor cells, only Ang-4 expression has prognostic impact in NSCLC. In tumor stroma, Ang-4 and Ang-2 are independently associated with survival. The prognostic impact of tumor cell VEGF-A in NSCLC appears strongly associated with a concomitantly high tumor cell expression of Ang-2.

## Introduction

Although lung cancer mortality in the western world is now declining, lung cancer still holds the position as the number one killer among cancers [1]. Effective novel therapies are warranted and angiogenesis is an attractive target [2], [3].

In the complex and dynamic process of angiogenesis, Angiopoietin (Ang)/Tie-2 receptor signaling has been shown to play a critical role in concert with vascular endothelial growth factor (VEGF) [4], [5]. Withdrawal of VEGF-A causes endothelial cells (ECs) lacking support of pericytes to undergo rapid apoptosis while ECs with associated mural cells expressing molecules involved in vascular remodeling (including Angs) survive [4], [6]–[8].

The three known human ligands for Tie-2 are Ang-1, Ang-2 and Ang-4. Ang-1 stimulates the kinase activity of Tie-2 upon binding. Ang-2 has been shown to act as a context-dependent antagonist or agonist for Tie-2 with the antagonism as the best described effect [9], [10]. Ang-4 is a ligand which seems to have the same agonistic effect on Tie-2 as Ang-1, but is less studied [11], [12]. Tie-2 downstream signaling most importantly mediates cell survival which in the vascular compartment maintains vascular quiescence, but also exerts anti-inflammatory effects. In NSCLC, the collaborating activities of Ang-2 and VEGF pathways have been suggested to promote tumor angiogenesis [13].

There are, however, conflicting reports on the role of the ang/Tie-2 axis. Under stimuli of Ang-1, Tie-2 signaling appears to mediate localization-specific effects as ECs with endothelial connections form Tie-2 bridges and reduces permeability and angiogenesis, while the same signaling in migrating ECs enhances motility and proliferation [14]. In addition, systemic treatment with an Ang-1 agonist in mice, has been shown to support tumor progression by increasing vascular entry of tumor cells leading to lung metastases [15].

As agents targeting the Ang/Tie-signaling pathway, alone or in combination with VEGF inhibition, are currently in phase I and phase II trials (www.clinicaltrials.gov), we aimed to evaluate the prognostic relevance of all angiopoietins, and their receptor Tie-2 in both tumor and stromal cells in a large unselected cohort of NSCLC patients. Based on the proposed interplay between VEGF-A and angiopoietins, it was also examined if angiopoietins influenced the prognostic impact of VEGF-A expression.

## Results

### Patient characteristics

The patients' demographic, clinical and histopathological data are presented in Table 1. The median follow-up time of survivors was 86 months (range 48–216). The median patient age was 67 (range 28–85) and 76% were male, 95% were in performance status 0–1 and 95% were present or previous smokers. The NSCLC tumors comprised 191 squamous cell carcinomas (SCC), 113 adenocarcinomas (AC) including 18 bronchioalveolar carcinomas (BAC) and 31 large-cell carcinomas (LCC).

**Table 1:** Patient characteristics and clinicopathological variables and their prognostic value for disease-specific survival in 335 NSCLC patients (univariate analyses; log rank test).

Characteristic	Patients(n)	Patients(%)	Median survival(months)	5-Year survival(%)	P
**Age**					
≤65 years	156	47	83	55	0.34
>65 years	179	53	NR	60	
**Sex**					
Female	82	25	190	63	0.20
Male	253	75	83	56	
**Smoking**					
Never	15	5	19	43	0.23
Current	215	64	NR	60	
Former	105	31	71	54	
**Performance status**					
PS 0	197	59	NR	63	**0.013**
PS 1	120	36	64	52	
PS2	18	5	25	33	
**Weight loss**					
<10%	303	90	127	58	0.71
>10%	32	10	98	57	
**Histology**					
SCC	191	57	NR	66	0.08
Adenocarcinoma	113	34	54	45	
LCC	31	9	98	56	
**Differentiation**					
Poor	138	41	47	47	**<0.001**
Moderate	144	43	190	64	
Well	53	16	NR	68	
**Surgical procedure**					
Lobectomy+Wedge*	243	73	190	61	**0.004**
Pneumonectomy	92	27	37	47	
**Pathological stage**					
I	157	47	190	71	**<0.001**
II	136	40	61	51	
IIIa	42	13	17	23	
**Tumor status**					**<0.001**
1	85	25	190	74	
2	188	56	84	57	
3	62	19	25	36	
**Nodal status**					
0	232	69	190	66	**<0.001**
1	76	23	35	43	
2	27	8	18	18	
**Surgical margins**					
Free	307	92	190	58	0.29
Not free	28	8	47	47	
**Vascular infiltration**					
No	284	85	190	58	**<0.001**
Yes	51	15	27	32	

*Wedge, n = 10.

Abbreviations: NR = not reached; PS = Performance status; SCC = Squamous cell carcinoma, LCC = Large-cell carcinoma.

### Expression of angiopoietins and correlations

Expressions of all the markers were mainly cytoplasmic. There was rare nuclear and membranous staining, except for Ang-4 which exclusively showed cytoplasmic staining. The staining was homogenous within cores except for Ang-2 and Tie-2 which had a small degree of heterogeneity.

When testing correlations between molecular markers and clinicopathological variables, we found high tumor cell expression of Ang-4 to correlate to histology (r = 0.19, P = 0.003), as it was expressed at a higher level in squamous cells. Between different molecular markers we found high tumor Ang-4 and Ang-1 expression to be moderately correlated (r = 0.18, P = 0.001). Further, high tumor cell Ang-2 expression correlated to high tumor cell VEGF-A expression (r = 0.15, P = 0.007).

### Univariate analyses

Results regarding the clinicopathological variables are presented in Table 1. WHO performance status (P = 0.013), differentiation (P<0.001), surgical procedure (P = 0.004), pathological stage (P<0.001), T-status (P<0.001), N-status (P<0.001) and vascular infiltration (P<0.001) were significant prognostic factors (Table 1).

**Figure 1:**
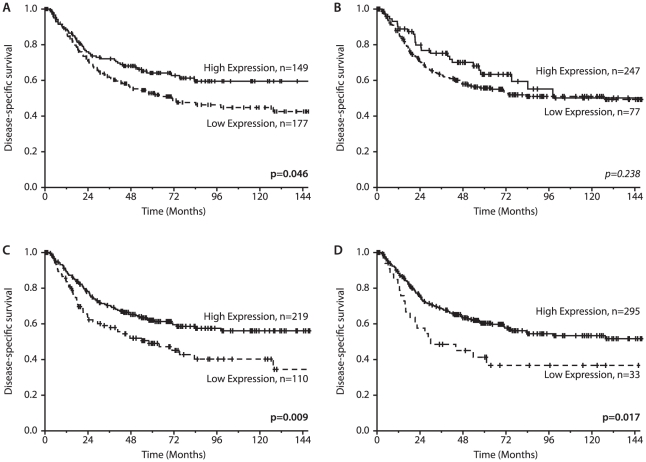
Disease-specific survival according to factor expression. Disease-specific Kaplan-Meier survival curves according to: A) Ang-4 expression in tumor cells, B) Ang-2 in tumor cells, C) Ang-4 in stromal cells and D) Ang-2 in stromal cells in resected NSCLC patients. The P-value is according to the log-rank test.

Data on the association between molecular markers and disease-specific survival (DSS), are given in Table 2 and Figure 1. High tumor cell expression of Ang-4 (P = 0.046) as well as high stromal cell expression of Ang-4 (P = 0.009) and Ang-2 (P = 0.017) were associated with a favorable DSS. For tumor cell Ang-2 expression alone there was no impact on survival (Figure 1b). The favorable impact of high tumor cell Ang-4 expression was most prominent for subgroups of patients below 65 years (P = 0.002), males (P = 0.027), squamous cell histology (P = 0.038), nodal status 1 (P = 0.007) and those without vascular infiltration (P = 0.015). For both Tie-2 and Ang-1 expression there was no association with DSS.

**Table 2:** Tumor cell and stromal markers as prognostic factors for disease-specific survival in 335 NSCLC patients (univariate analyses; log-rank test).

Characteristics	Patients (n)	Patients (%)	Median survival (months)	5-year survival (%)	P
**Ang-1**					
**Tumor**					0.150
High	183	55	190	63	
Low	141	42	84	52	
Missing	11	3			
**Stroma**					
High	237	71	190	62	0.096
Low	92	27	58	50	
Missing	6	2			
**Ang-2**					
**Tumor**					0.238
High	77	23	NR	63	
Low	247	74	127	56	
Missing	11	3			
**Stroma**					**0.017**
High	295	88	190	60	
Low	33	11	30	41	
Missing	7	2			
**Ang-4**					
**Tumor**					**0.046**
High	149	44	190	64	
Low	177	53	71	53	
Missing	9	3			
**Stroma**					**0.009**
High	219	65	190	62	
Low	110	33	58	49	
Missing	6	2			
**Tie2**					0.267
**Tumor**	182	54	98	56	
High	140	42	NR	60	
Low	13	4			
Missing					
**Stroma**					0.116
High	58	17	NR	69	
Low	269	80	98	56	
Missing	8	3			
**VEGFA and Ang-2** *					
**Low Ang-2**	247				0.078
High VEGF-A	117	35	64	50	
Low VEGF-A	130	39	190	61	
**High Ang-2**	77				**<0.001**
High VEGF-A	23	7	30	32	
Low VEGF-A	54	16	NR	78	
Missing	11	3			

*Tumor data.

**Figure 2:**
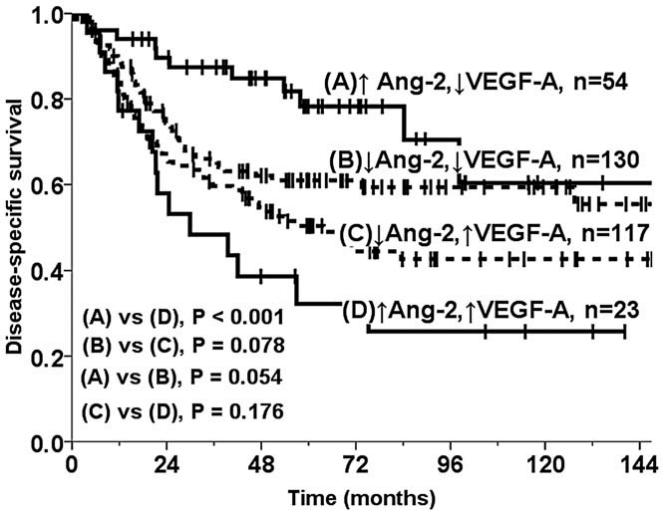
Disease-specific survival according to co-expression of Ang-2 and VEGF-A. Disease-specific Kaplan-Meier survival curves according to the co-expression of VEGF-A and Ang-2 in resected NSCLC patients. The P-value is according to the log-rank test.

There was a profound survival impact of high tumor cell VEGF-A expression, but only in patients with concomitantly high Ang-2 tumor cell expression (Table 2, P<0.001, Figure 2). At low Ang-2 expression, tumor cell expression of VEGF-A had an insignificant prognostic impact (P = 0.078). Detailed results regarding VEGF-A expression data have been published earlier [16].

### Multivariate analyses

Results of the multivariate analysis are presented in Table 3. In model 1, where all patients were assessed, high stromal Ang-4 (HR = 1.47, CI 95% 1.02–2.11, P = 0.04), stromal Ang-2 expression (HR = 1.88, CI 95% 1.15–3.08, P = 0.012) and high tumor cell expression of VEGF-A (HR = 1.49, CI 1.04–2.14, P = 0.029) were significant independent prognosticators for DSS in addition to several clinicopathological variables (tumor status, P<0.001: nodal status, P<0.001; performance status, P = 0.013; vascular infiltration; P = 0.011; differentiation, P = 0.033). High tumor cell expression of Ang-4 did not, however, reach statistical significance (P = 0.15).

**Table 3:** Results of Cox regression analyses (Backward stepwise model).

	Model 1 (All patients)			Model 2 (Patients with high Ang-2 expression)		
Factor	HazardRatio	95% CI	P	HazardRatio	95% CI	P
**Tumor status**			<0.001*			NS
1	1.00			NS		
2	1.72	1.04–2.85	0.034	NS	NS	NS
3	3.25	1.83–5.75	<0.001	NS	NS	NS
**Nodal status**			<0.001*			0.003*
0	1.00			1.00		
1	1.82	1.20–2.76	0.005	3.93	1.57–9.81	0.003
2	2.88	1.68–4.92	<0.001	13.9	1.09–177	0.042
**Performance status**			0.01*			0.024*
ECOG 0	1.00			1.00		
ECOG 1	1.78	1.22–2.60	0.003	3.42	1.22–9.62	0.02
ECOG 2	NS	NS	NS	4.03	1.06–15.5	0.04
**Vascular infiltration**			0.011			NE
No	1.00			NE		
Yes	1.85	1.15–2.97		NE	NE	
**Differentiation**			0.023*			0.034
Poor	1.00			1.00		
Moderate	NS	NS	NS	NS	NS	NS
Well	NS	NS	NS	0.05	0.005–0.5	0.01
**Ang-2 stroma**			0.018			NE
Low	1.81			NE		
High	1	1.11–2.96		NE	NE	
**Ang-4 stroma**			0.033			NE
Low	1.49			NE		
High	1	1.03–2.16		NE	NE	
**Ang-4 tumor**			NS			NE
Low	NS			NE		
High	NS	NS		NE	NE	
**VEGF-A tumor**			0.029			<0.001
Low	1.00			1.00		
High	1.49	1.04–2.14		6.43	2.46–16.8	

*Overall significance as a prognostic factor.

NE = not entered in the analysis. NS = Not significant. In model 2 vascular infiltration was not entered due to non-significance in univariate analysis in this subgroup.

In model 2, only patients with high tumor cell expression of Ang-2 were assessed (N = 88, Table 3). In this subgroup, high tumor cell expression of VEGF-A mediated an independent negative prognostic effect (HR = 6.43, CI 95% 2.46–16.79, P<0.001), in addition to positive nodal status (P = 0.003), reduced performance status (P = 0.024) and poor differentiation (P = 0.034).

## Discussion

We present a large-scale study in an unselected population of surgically resected NSCLC patients using high-throughput TMA. In tumor cells, Ang-4 was the only angiopoietin to be associated with survival, although not in an independent fashion. We found that low stromal cell expressions of Ang-4 and Ang-2 were independent poor prognostic factors for survival. VEGF-A was a powerful poor prognosticator in patients with high tumor cell Ang-2 expression, but not in those with low expression.

To our knowledge, this is the first prognostic evaluation of Ang-4 expression in any human cancer. An improved prognosis following high tumor cell expression of Ang-4 is in accordance with an earlier functional *in vitro* study. Olsen and co-workers found [12] Ang-4 to inhibit angiogenesis and reduce the elevated interstitial pressure induced by basic fibroblast growth factor (bFGF) and VEGF in small cell lung cancer tumor cells (GLC19). However, recently Brunckhorst and colleagues found Ang-4 to promote glioblastoma progression *in vitro* by enhancing tumor cell viability and angiogenesis [17].

In the present study, there was no association between tumor cell expression of Ang-2, Tie-2 and survival. Earlier IHC-studies in NSCLC have shown conflicting results. In the largest study of 236 NSCLC cases, Tanaka et al. found that high tumor cell Ang-2 expression was significantly associated with poor overall survival in the multivariate analysis [18]. They found that high tumor cell Ang-2 expression was significantly associated with poor overall survival even in the multivariate analysis. Corroborating our results, they observed that the co-expression of Ang-2 and VEGF resulted in a particularly poor survival. Due to their heterogeneous staining intensity and low expression in endothelial cells, they were not able, however, to semiquantitatively evaluate the degree of Ang-1 or Ang-2 expression or to distinguish positive or negative expression in endothelial cells. This is in contrast to the experience by us and others [19], [20]. In a smaller NSCLC study, Reinmuth et al. found that high tumor cell Ang-1 expression, combined intensity and percent of positive tumor cells, was statistically associated to poor survival. Takanami investigated mRNA expression of Ang-2 in 77 operable NSCLC patients and found that a high expression was independently associated with a poor survival [21]. High Ang-2 expression has also been associated with poor prognosis in oral [22], colorectal [20] and bladder cancer [23] whereas Sie et al. found the Ang-1/Ang-2 balance to be associated to a poor survival in glioblastoma multiforme [24]. These conflicting results may be due to variations in methods, end-points, patient selection and/or cut-off levels.

In stroma, on the other hand, there are no previous studies examining the prognostic impact of these markers. This is surprising since angiogenesis definitely also involves endothelial cells and surrounding stromal cells. Attention should be paid to the different tumor compartments. To illustrate this, the expression of immunological markers in NSCLC impacts survival differentially in tumor and stroma [25]–[27]. Herein, systematic studies of tumor vessels could not be performed due to the fact that we had only two stromal cores of 0.6 mm available from each patient. Nevertheless, the expression of Ang/Tie-2 markers has been investigated in the stromal compartments where the cross-talk between endothelial cells, fibroblasts, immunological cells and tumor cells are vital for angiogenesis [28]. Consistently, both Ang-4 and Ang-2 expression in stroma proved to be independently associated with an improved survival. Since these markers are known to exert opposite effects upon binding to Tie-2, the similar beneficial prognostic effect remains to be elucidated.

Since the angiogenic effect of the angiopoietin system is strongly linked to VEGF we examined the prognostic effects of co-expressions of angiopoietins and VEGF-A co-expressions in tumor. High tumor cell Ang-2 expression alone had no prognostic survival impact, but when co-expressed with a high rather than low VEGF-A level, this combination led to a significantly worse 5-year survival (32%) with an HR at 6.43. Besides, patients with a concomitantly low VEGF-A expression and high Ang-2 expression in tumor cells tended to a better survival compared with those with a low tumor cell Ang-2 expression. These results can be explained by the functional role of Ang-2, as it is known to destabilize the endothelium, and the plastic state triggered by Ang-2 can lead to new vessel growth or vessel regression, depending on the presence of factors such as VEGF-A [4], [28]. At low levels of VEGF-A, high Ang-2 levels may lead to vessel regression and a better prognosis. This is in accordance with a study by Huang and colleagues [8] who detected that both overexpression of Ang-1 and administration of an Ang-1 agonist, induced a shift towards Tie-2 stimulation and protected tumors and vasculature from regression.

Further, we observed that VEGF-A expression had a survival impact, but only in patients with high Ang-2 expression. It may be speculated if cancers with a high tumor cell Ang-2 expression are more susceptible to anti-VEGF-A treatment (e.g. bevacizumab). Hitherto, there are no studies evaluating the effect of bevacizumab retrospectively or in a stratified manner according to Ang-2 expression. This may be an interesting approach.

In conclusion, only the expression of the angiopoietin Ang-4 in tumor cells had impact on NSCLC survival. In the stroma, however, Ang-2 and Ang-4 expression was independently associated with an improved survival although the biological rationale for these stroma results remains unclear. The intriguing results from the Ang-2 expression subgroups where the negative prognostic impact of high tumor cell VEGF-A expression was highly dependent on tumor cell expression of Ang-2, must be cautiously interpreted as the high Ang-2 subgroups were small. These results however raises the hypotheses that there might be potential benefits of dual targeting of Ang-2 and VEGF-A and that Ang-2 biomarker assessments in VEGF targeted trials might identify important subgroups.

## Materials and Methods

### Patients

Primary tumor tissues, from all the 371 patients surgically resected for pathological stage I to IIIA NSCLC at the University Hospital of North Norway and Nordland Central Hospital from 1990 to 2004, were retrospectively identified in the archives of the two pathological departments. A total of 36 patients were excluded from the study due to inadequate paraffin-embedded fixed tissue blocks (n = 13), other malignancy within the 5 years prior to diagnosis (n = 13) or having received radiotherapy or chemotherapy prior to surgery (n = 10). Preoperative chemotherapy was not considered standard treatment in Norway during the actual period. Complete demographic and clinicopathological data for these 335 eligible patients were obtained retrospectively. The pathological data were revised according to the 7^th^ edition of UICC TNM classification of lung cancer [29], [30]. The last DSS update was done in November 2008.

### Tissue Microarray construction

Duplicate 0.6 mm core biopsies from the most representative areas of tumor cells (neoplastic epithelial cells) and tumor stroma were collected from each surgical specimen using a tissue-arraying instrument (Beecher Instruments, Silver Springs, MD). Normal lung tissue localized distant from the primary tumor as well as lung tissue samples from 20 normal lungs were used as controls. Eight tissue microarray blocks (TMAs) were constructed to include all the cores. The detailed methodology has been reported previously [16].

### Immunohistochemistry

The applied commercial antibodies had been subjected to in-house validation by the manufacturer for IHC on paraffin-embedded material (IHC-P). One exception was Ang-1 which was selected due to others' published success with this antibody [18], [20], [22], [31], [32] and failure to achieve satisfying quality with other available IHC-P tested antibodies. The 4 µm TMA sections were deparaffinised with xylene and rehydrated with ethanol. The sections containing tissue cores were subjected to the following antibodies: Ang-1 (goat polyclonal, sc-6319, Santa Cruz biotechnology, Inc., 2 µg/ml), Ang-2 (rabbit, polyclonal, ab65835, Abcam, 17 µg/ml), Ang-4 (goat polyclonal, AF964, R&D systems, 4 µg/ml), Tie-2 (rabbit polyclonal, sc-9026, Santa Cruz biotechnology, Inc., 4 µg/ml) and VEGF-A (rabbit polyclonal; RB-1678; Neomarkers; 100 µg/ml).

Antigen retrieval was done manually for all antibodies except Ang-2, by placing the specimens in 0.01 M citrate buffer at pH 6.0 and exposed to microwave heating of 20 minutes at 450 W. All antibodies were incubated overnight at 4°C except for VEGF-A where the primary antibody was incubated for 30 min in room temperature. For Ang-2 we used the Ventana Benchmark XT® (Ventana Medical Systems Inc.), procedure ultraview DAB v3 with automatic antigen retrieval with CC1 mild (30 min). Negative controls were simultaneously performed for all antibodies by omitting the primary antibody and an appropriate isotype-control was done for all antibodies on one of the TMA slides. Capillary vessels in stromal cores with high expression were used for internal positive controls and skin haemangiomas as external positive controls

Finally, all slides were counterstained with hematoxylin to visualize the nuclei.

### Scoring of immunohistochemistry

**Figure 3:**
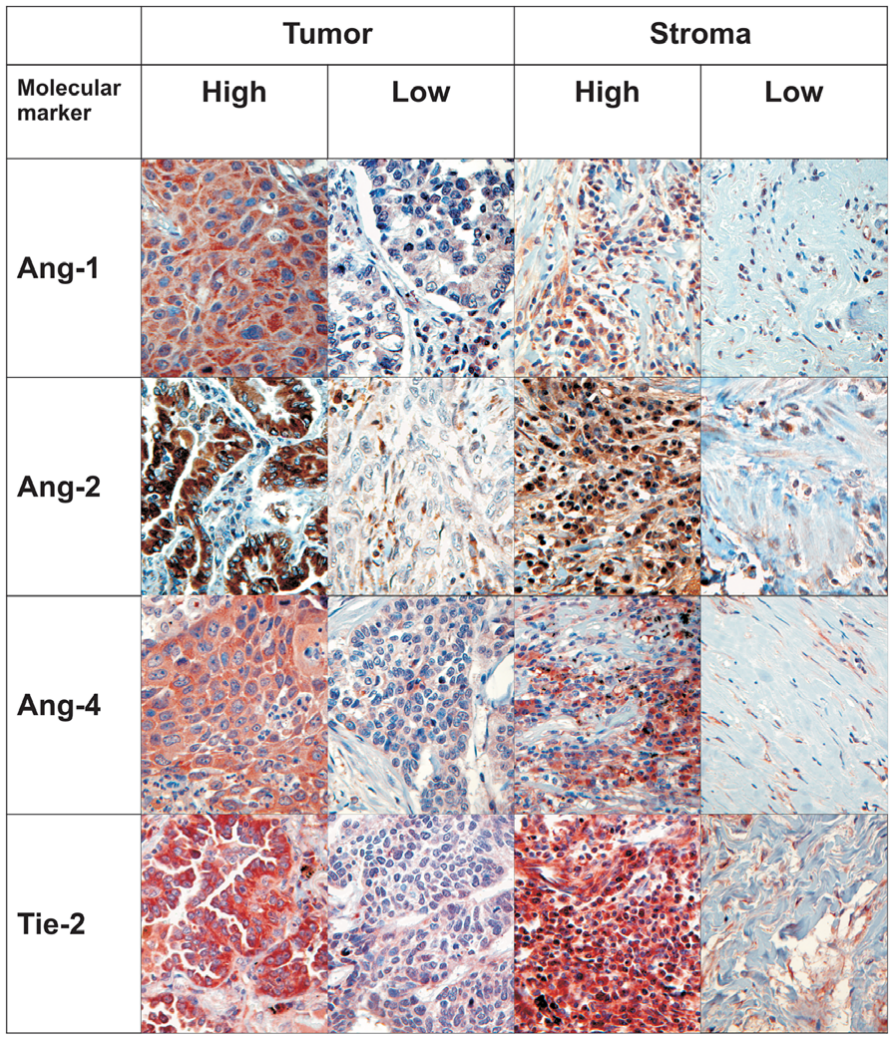
Immunohistochemical staining. Immunohistochemical analyses of NSCLC representing high and low intensities for tumor cell and stromal expression of Ang-1, Ang-2, Ang-4 and Tie-2.

Each anonymized core was scored independently and semiquantitatively by two pathologists (S.A.S and K.A.S) by light microscopy. Only viable parts were scored and even though some cores comprised of both tumor and stroma, only one entity was scored at a time. In stroma, we scored all non-neoplastic cells. The dominant staining intensity in both tumor and stroma cores was scored as: 0 = negative, 1 = weak, 2 = intermediate and 3 = strong (Figure 3). Only cytoplasmic staining was evaluated. In stroma cores the cytoplasmic staining was scored with regards to density as well: 1 = low, 2 = intermediate and 3 = high.

In case of disagreement the slides were re-examined and consensus was reached by the observers. Interindividual variability with respect to IHC-scoring in both tumor cells and stromal cells was evaluated on the current material in a previous paper (r = 0.95, range 0.93–0.98) [16].

A mean score was calculated separately for the two tumor cell cores and the two stromal cells cores in each individual. In tumor, high expression was defined as ≥2.0 for Ang-1, Ang-4 and Tie-2, and ≥2.5 for Ang-2. In stroma, the sum of mean density (1–3) and intensity score (0–3) was calculated. High expression in stroma was defined as ≥2.5 for Ang-1, Ang-2 and Ang-4, and ≥5.0 for Tie2. Details regarding VEGF-A scoring has been reported earlier [16]. Similar scoring methods have been used in our previous IHC-scoring studies [16], [27], [33] and by others [34].

### Statistical methods

The statistical analyses were done using the SPSS 16.0 package (Chicago, IL). The χ2 test and Fishers exact tests were used to examine the associations between molecular marker expressions and the clinicopathological markers. Plots of the DSS, according to marker expressions, were drawn using the Kaplan-Meier method, and statistical significance between survival curves was assessed by the log rank test. The survival curves were terminated at 146 months, due to less than 10% of patients at risk after this point. The chosen endpoint, DSS, was calculated from the time of surgery to the time of lung cancer death. All significant variables from the univariate analyses were entered into the multivariate analyses in a backward stepwise Cox regression analysis with a probability for stepwise entry and removal at 0.05 and 0.10, respectively. A P<0.05 was considered statistically significant for all analyses.

### Ethics

The Norwegian Data Inspection Board and The Regional Committee for research ethics have approved the study. Information and subsequent written consent from patients was considered, but as this was a retrospective study with more than half of patients deceased, the rest of the patients having to reminded about the death rate of the disease and the possible raising of unrealistic hope for the individual, they specifically waived the need for consent.

## References

[1] Jemal A, Siegel R, Ward E, Hao Y, Xu J (2009). Cancer statistics, 2009.. CA Cancer J Clin.

[2] Mantovani A (2009). Cancer: Inflaming metastasis.. Nature.

[3] Hanahan D, Weinberg RA (2000). The hallmarks of cancer.. Cell.

[4] Saharinen P, Bry M, Alitalo K (2010). How do angiopoietins Tie in with vascular endothelial growth factors?. Curr Opin Hematol.

[5] Fukuhara S, Sako K, Noda K, Zhang J, Minami M (2010). Angiopoietin-1/Tie2 receptor signaling in vascular quiescence and angiogenesis.. Histol Histopathol.

[6] Benjamin LE, Golijanin D, Itin A, Pode D, Keshet E (1999). Selective ablation of immature blood vessels in established human tumors follows vascular endothelial growth factor withdrawal.. J Clin Invest.

[7] Puri MC, Rossant J, Alitalo K, Bernstein A, Partanen J (1995). The receptor tyrosine kinase TIE is required for integrity and survival of vascular endothelial cells.. EMBO J.

[8] Huang J, Bae JO, Tsai JP, Kadenhe-Chiweshe A, Papa J (2009). Angiopoietin-1/Tie-2 activation contributes to vascular survival and tumor growth during VEGF blockade.. Int J Oncol.

[9] Augustin HG, Koh GY, Thurston G, Alitalo K (2009). Control of vascular morphogenesis and homeostasis through the angiopoietin-Tie system.. Nat Rev Mol Cell Biol.

[10] Sato TN, Tozawa Y, Deutsch U, Wolburg-Buchholz K, Fujiwara Y (1995). Distinct roles of the receptor tyrosine kinases Tie-1 and Tie-2 in blood vessel formation.. Nature.

[11] Lee HJ, Cho CH, Hwang SJ, Choi HH, Kim KT (2004). Biological characterization of angiopoietin-3 and angiopoietin-4.. FASEB J.

[12] Olsen MW, Ley CD, Junker N, Hansen AJ, Lund EL (2006). Angiopoietin-4 inhibits angiogenesis and reduces interstitial fluid pressure.. Neoplasia.

[13] Wong MP, Chan SY, Fu KH, Leung SY, Cheung N (2000). The angiopoietins, tie2 and vascular endothelial growth factor are differentially expressed in the transformation of normal lung to non-small cell lung carcinomas.. Lung Cancer.

[14] Saharinen P, Eklund L, Miettinen J, Wirkkala R, Anisimov A (2008). Angiopoietins assemble distinct Tie2 signalling complexes in endothelial cell-cell and cell-matrix contacts.. Nat Cell Biol.

[15] Holopainen T, Huang H, Chen C, Kim KE, Zhang L (2009). Angiopoietin-1 overexpression modulates vascular endothelium to facilitate tumor cell dissemination and metastasis establishment.. Cancer Res.

[16] Donnem T, Al-Saad S, Al-Shibli K, Delghandi MP, Persson M (2007). Inverse prognostic impact of angiogenic marker expression in tumor cells versus stromal cells in non small cell lung cancer.. Clin Cancer Res.

[17] Brunckhorst MK, Wang H, Lu R, Yu Q (2010). Angiopoietin-4 promotes glioblastoma progression by enhancing tumor cell viability and angiogenesis.. Cancer Res.

[18] Tanaka F, Ishikawa S, Yanagihara K, Miyahara R, Kawano Y (2002). Expression of angiopoietins and its clinical significance in non-small cell lung cancer.. Cancer Res.

[19] Reinmuth N, Piegelbrock E, Raedel M, Fehrmann N, Hintelmann H (2007). Prognostic significance of vessel architecture and vascular stability in non-small cell lung cancer.. Lung Cancer.

[20] Chung YC, Hou YC, Chang CN, Hseu TH (2006). Expression and prognostic significance of angiopoietin in colorectal carcinoma.. J Surg Oncol.

[21] Takanami I (2004). Overexpression of Ang-2 mRNA in non-small cell lung cancer: association with angiogenesis and poor prognosis.. Oncol Rep.

[22] Chien CY, Su CY, Chuang HC, Fang FM, Huang HY (2008). Angiopoietin-1 and -2 expression in recurrent squamous cell carcinoma of the oral cavity.. J Surg Oncol.

[23] Szarvas T, Jager T, Totsch M, vom DF, Kempkensteffen C (2008). Angiogenic switch of angiopietins-Tie2 system and its prognostic value in bladder cancer.. Clin Cancer Res.

[24] Sie M, Wagemakers M, Molema G, Mooij JJ, de Bont ES (2009). The angiopoietin 1/angiopoietin 2 balance as a prognostic marker in primary glioblastoma multiforme.. J Neurosurg.

[25] Welsh TJ, Green RH, Richardson D, Waller DA, O'Byrne KJ (2005). Macrophage and mast-cell invasion of tumor cell islets confers a marked survival advantage in non-small-cell lung cancer.. J Clin Oncol.

[26] Wakabayashi O, Yamazaki K, Oizumi S, Hommura F, Kinoshita I (2003). CD4+ T cells in cancer stroma, not CD8+ T cells in cancer cell nests, are associated with favorable prognosis in human non-small cell lung cancers.. Cancer Sci.

[27] Al-Shibli KI, Donnem T, Al-Saad S, Persson M, Bremnes RM (2008). Prognostic effect of epithelial and stromal lymphocyte infiltration in non-small cell lung cancer.. Clin Cancer Res.

[28] Huang H, Bhat A, Woodnutt G, Lappe R (2010). Targeting the ANGPT-TIE2 pathway in malignancy.. Nat Rev Cancer.

[29] Goldstraw P (2009). The 7th Edition of TNM in Lung Cancer: what now?. J Thorac Oncol.

[30] World Health Organization (1999). Histological Typing of Lung and Pleural Tumours.

[31] Nakayama T, Yoshizaki A, Kawahara N, Ohtsuru A, Wen CY (2004). Expression of Tie-1 and 2 receptors, and angiopoietin-1, 2 and 4 in gastric carcinoma; immunohistochemical analyses and correlation with clinicopathological factors.. Histopathology.

[32] Nakayama T, Inaba M, Naito S, Mihara Y, Miura S (2007). Expression of angiopoietin-1, 2 and 4 and Tie-1 and 2 in gastrointestinal stromal tumor, leiomyoma and schwannoma.. World J Gastroenterol.

[33] Al-Saad S, Al-Shibli K, Donnem T, Persson M, Bremnes RM (2008). The prognostic impact of NF-kappaB p105, vimentin, E-cadherin and Par6 expression in epithelial and stromal compartment in non-small-cell lung cancer.. Br J Cancer.

[34] Soltermann A, Tischler V, Arbogast S, Braun J, Probst-Hensch N (2008). Prognostic significance of epithelial-mesenchymal and mesenchymal-epithelial transition protein expression in non-small cell lung cancer.. Clin Cancer Res.

